# RNA Modifications Modulate Biomolecular Condensates in Stress and Disease

**DOI:** 10.3390/genes17080973

**Published:** 2026-08-19

**Authors:** Y. Sprecher, M. Sevilla-Sharon, S. Moshitch-Moshkovitz

**Affiliations:** 1 Sheba Cancer Research Center, Sheba Medical Center, Tel Hashomer, Ramat Gan 5266202, Israel; sprecher@mail.tau.ac.il (Y.S.); michal.sevilla-sharon@weizmann.ac.il (M.S.-S.); 2 Gray Faculty of Medical and Health Sciences, Tel Aviv University, Tel Aviv 6997801, Israel

**Keywords:** biomolecular condensates (BMCs), RNA modifications, epitranscriptome, liquid-liquid phase separation (LLPS), *N*^6^-methyladenosine (m^6^A), *N*^1^-methyladenosine (m^1^A), 5-methylcytidine (m^5^C), multivalency

## Abstract

Biomolecular condensates are dynamic membraneless organelles composed of proteins and RNAs that assemble through multivalent interactions and provide cells with powerful means to regulate gene expression in space and time. Different epitranscriptomic marks such as m^6^A, m^1^A, and m^5^C can reshape RNA structure—binding interfaces and multivalency and, in this manner, tuning which transcripts nucleate or partition into specific condensates and influencing their material state. This review summarizes how individual RNA modifications and their associated proteins regulate the formation and function of BMCs such as stress granules, P-bodies, nuclear bodies and disease-linked condensates in cancer and neurodegeneration. It highlights emerging concepts of combinatorial “epitranscriptomic codes” and bidirectional feedback between condensates and RNA-modifying enzymes and discusses the current experimental and technical gaps that still limit our understanding of modification crosstalk and condensate topology.

## 1. Introduction

Eukaryotic cells contain membrane-bound organelles surrounded by lipid bilayers, along with a rich repertoire of membraneless compartments, collectively termed biomolecular condensates (BMCs). These compartments are found both in the nucleus and the cytoplasm and composed mainly of RNAs and proteins that can condense and dissolve in response to changing physiological conditions, stress and diverse cellular cues through phase separation [1,2,3]. While BMCs can form by liquid–liquid phase separation (LLPS), it is important to recognize that it is not always the dominant mechanism, other assembly mechanisms such as scaffolding, polymerization, and higher-order oligomerization contribute to the formation of membraneless compartments [4,5]. The levels of evidence in this review are explained in Box 1.

Phase separation is driven predominantly by multivalent interactions between and within proteins and RNA molecules, which are not merely passive cargo but also act as key structural and regulatory elements, actively affecting nucleation, scaffolding and condensate architecture [6]. These multivalent networks confer tunable material properties to BMCs, allowing them to span a range of states from highly dynamic liquid-like droplets to more viscoelastic, gel-like forms. Thus, phase-separated condensates provide cells with a means for rapid and dynamic control through spatiotemporal molecular interactions, enabling effective adaptation to changing conditions [7,8].

RNA modifications, termed the epitranscriptome, add a powerful regulatory layer that alters RNA fate without changing its sequence [9]. Epitranscriptomic marks such as *N*^6^-methyladenosine (m^6^A), *N*^1^-methyladenosine (m^1^A) and 5-methylcytidine (m^5^C, commonly referred to in the RNA-modification literature as 5-methylcytosine), can modulate the physicochemical behavior of transcripts. These modifications can alter base-pairing and RNA folding, burying or exposing binding sequences [10,11], and consequently affecting the recruitment of specific reader proteins [10,11].

These effects have the potential to affect the material properties of condensates, including their assembly, molecular exchange dynamics, viscosity, and composition. Modification-induced restructuring effectively changes the interaction profiles of modified transcripts, which can lead RNAs toward or away from particular BMCs, influencing their formation, composition and dynamics. However, direct evidence is strongest for m^6^A- and m^1^A-dependent recruitment of phase-separating reader proteins, whereas the condensate-regulatory roles of other RNA modifications remain less characterized [10].

In this review we outline the principles of condensate formation from both RNA- and protein-centered perspectives and discuss how specific RNA modifications shape the assembly and function of key BMCs. Several recent reviews have discussed RNA modifications in the context of phase separation and condensate biology [10,12,13,14,15,16]. However, most have focused either on a single modification, a limited subset of condensates, or one disease area. In contrast, the present review aims to provide an evidence-graded synthesis across epitranscriptomic marks that currently have direct or supportive links to BMCs, primarily m^6^A, m^1^A and m^5^C, covering both nuclear and cytoplasmic bodies and their roles in stress adaptation, cancer and neurodegeneration. We offer our perspectives regarding emerging concepts such as combinatorial “epitranscriptomic codes” in regulation of BMCs, explore the roles of condensates in neurodegeneration, cancer, and stress adaptation and suggest how these interactions may be utilized as a therapeutic axis. This review offers an RNA-centered biophysical framework, which examines how RNA modifications alter transcript structure, valency, and scaffold behavior to shape condensate identity, composition, and material properties.

Box 1 **Levels of evidence used in this review.**

  We use BMC as a general term for membraneless assemblies enriched in specific proteins and RNAs, and reserve “phase separation” and “liquid-like” for cases showing the hallmarks of LLPS. We grade the evidence for each example as direct (in vitro phase behavior, or cellular condensation with LLPS-focused perturbations), supportive (partitioning into a preexisting condensate without direct LLPS tests), or indirect/speculative (altered puncta number, localization, enzyme expression or disease state, with no demonstrated causal change in phase behavior). We state which category each key study falls into (see Table 1) and follow current community recommendations for the rigorous study of condensates [17].

**Table 1:** Evidence-graded data of RNA modifications linked to biomolecular condensates.

	Writer	Eraser	Reader/RBP	BMC	Model	Evidence Type	Direct PS Assay	Functional Consequence	Disease	Primary Ref.	Principal Limitation	Grade
m^6^A	METTL3 METTL14	FTO ALKBH5	YTHDF1-3	SGs P-bodies	Oxidative + heat stress; HeLa, U2OS	In vitro droplets + LLPS-focused perturbations (domain swap, YTH mutants)	✓	Poly-m^6^A promotes YTHDF condensation and SG/P-body assembly	-	[18,19,20]	Bulk assembly vs. single-transcript partitioning differs	D
	METTL3 METTL14	-	endogenous (none tested)	SGs	Endogenous transcripts; m^6^A depletion	Single-transcript partitioning; loss-of-m^6^A	✗	Loss of m^6^A has limited effect on partitioning of endogenous mRNAs	-	[21]	Counter-evidence; context-dependent	S*
	METTL3 METTL14	-	YTHDC1	Nuclear nYACs	AML cells	Cellular LLPS; IDR/YTH mutants disrupt condensation	✓	Protects leukemogenic transcripts from PAXT; leukemia maintenance	AML	[22,23]	Cell-type specific	D
	METTL3	-	YTHDC1 + BRD4	Enhancer transcriptional condensates	MCF7 breast cancer	Cellular LLPS; YTHDC1 loss reduces PS	✓	m^6^A-eRNA-YTHDC1-BRD4 condensates activate oncogenic transcription	Breast cancer	[23]	-	D
	METTL3 METTL14	-	YTHDF2	Cytoplasmic YTHDF2 condensates	Arsenite + PM_2.5_; keratinocyte, lung	PS-defective mutants blunt effect	✓	Represses PTEN/GADD45A translation; transformation	Lung, skin cancer	[24,25]	-	D
	METTL3 METTL14	-	IGF2BP1-3	mRNP granules SGs	Osmotic & oxidative stress	In vitro PS; PTM tuning; SGs dispensable for stabilization	~	Stabilizing hubs for target mRNAs	Cancer	[26,27,28]	SGs dispensable for stabilization; PS requirement unclear	S
	METTL3 METTL14	-	hnRNPC hnRNPG (m^6^A switch)	-	In vitro + cellular	Structural switch; indirect	✗	Exposes buried motifs; tunes RBP binding	-	[29,30]	No direct condensate assay	Sp
m^1^A	TRMT6/TRMT61A	-	no defined reader	SGs	Heat shock	Writer relocalization + reporter partitioning	✗	m^1^A-tagged mRNAs routed to SGs and protected	-	[31]	Localization ≠ catalytic change in granule	S
	- (on CAG-repeat RNA)	-	TDP-43	Cytoplasmic TDP-43 condensates	Neuronal CAG-repeat models	In vitro + cellular; m1A alters TDP-43 material state	✓	Drives TDP-43 translocation; less-dynamic condensates	ALS/FTD	[32]	Specific repeat-RNA context	D
	TRMT6/TRMT61A	ALKBH3	YTHDF1	PML nuclear bodies	Ocular melanoma	Condensate abundance via SP100A level	✗	m^1^A-SP100A axis controls PML-body abundance	Melanoma	[33]	Abundance not PS; YTHDF1-m^1^A reading not general	S
	TRMT6	-	YTHDF3	no BMC demonstrated	Lung SCC (LUSC)	Stability/proliferation	✗	Stabilizes cell-cycle mRNAs (TOPBP1, DSN1)	LUSC		No condensate link shown	Sp
m^5^C	NSUN2	-	YBX1	mRNP P-body-like granules exosome sorting	Cells	In vitro + cellular; IDR mutants reduce PS	✓	m^5^C-dependent mRNA stabilization; miRNA sorting	Ovarian & other cancers	[34,35,36]	miRNA-sorting m^5^C-dependence unclear	D
	NSUN family	-	YBX2	Cytoplasmic mRNP granules	In vitro + cells	In vitro PS; m^5^C lowers threshold; binding-pocket mutant	✓	m^5^C-dependent scaffold for selected mRNAs	-	[37]	-	D
	NSUN2	-	ALYREF	Nuclear export bodies	-	Association only	✗	m^5^C reader in nuclear export	-	[38,39]	Not mechanistically linked to PS	Sp
Other marks	ADAR1/2; METTL1; PUS	-	-	Various	-	Conceptual indirect	✗	Alter RNA structure and RBP recognition	-	[5,40,41,42,43]	No direct PS or material-state assay	Sp

Grade: D = direct; S = supportive; S* = supportive (counter-evidence); Sp = speculative; Direct PS assay: ✓ yes, ~ partial, ✗ no; PS = phase separation; SGs = stress granules; Other marks = A-to-I editing, m^7^G, pseudouridine (Ψ).

## 2. Principle of Biomolecular Condensate Formation

BMCs are widely distributed throughout the nucleus and cytoplasm playing central roles in diverse cellular processes, including RNA metabolism, ribosome biogenesis, genome maintenance, and signaling [1]. Table 2 and Table 3 summarize the main role of the key BMCs in the nucleus and the cytoplasm of human cells. Studying BMCs has encountered technical obstacles that stalled their characterization. What makes BMCs unique organelles? Unlike eukaryotic membrane-bound organelles, BMCs can granulate and dissolve dynamically, forming a spatiotemporally organized regulatory network to enable coordinated control of gene expression programs and cellular stress responses [1].

Condensate assembly is driven by a combination of cis- and trans-acting factors among their components. Trans-acting features are typically embedded within proteins and include structural domains that mediate interactions with RNA or with other proteins. They include features such as binding pockets and modular interaction domains. These features often underpin protein family classification [44]. Cis-acting elements are encoded within RNA molecules and encompass specific nucleotide sequences, RNA structures, and RNA modifications that together influence recruitment into condensates [5,9]. This definition may be simplified by considering cis-acting factors as encoded in the RNA itself, while trans-acting factors are found in molecules that act on the RNA.

**Table 2:** BMCs in the human cell nucleus [45].

BMC	Function	Size (Diameter)	Key Markers	Representative RNAs
Nucleoli	Major hub for ribosome biogenesis, coordinating rRNA transcription, processing and ribosomal subunit assembly; also acts as a sensor of cellular stress influencing cell-cycle and apoptosis.	0.5–2 µm	NCL; NPM1; FBL	rRNAs; Small nucleolar RNAs (snoRNAs)
Nuclear speckles (NSs)	Reservoirs for pre-mRNA splicing and processing factors that couple transcription, splicing and mRNA export, enhancing RNA maturation at highly transcribed genes.	0.5–5 µm	SRSF2 (SC35); SRRM2; SON	Spliceosomal snRNAs; poly(A)^+^ RNAs; pre-mRNAs; MALAT1
Cajal bodies (CBs)	Sites for small nuclear and small nucleolar RNPs biogenesis and modification, and for assembly of telomerase and other ribonucleoprotein complexes.	0.3–1 µm	COIL; SMN	Spliceosomal snRNAs, scaRNAs, telomerase RNA (TERC)
Paraspeckles	Regulate nucleocytoplasmic transport and translation by retaining A-to-I-edited and other regulatory mRNAs, and modulate transcription by sequestering factors such as SFPQ, especially under stress.	0.2–0.5 µm	NEAT1; SFPQ; PSPC1; TDP-43; NONO	NEAT1 (both isoforms); Retained A-to-I edited mRNAs; stress-responsive mRNAs
Promyelocytic leukemia protein (PML) bodies	Nuclear hubs for proteostasis and genome maintenance, coordinating SUMO-dependent protein modification, DNA damage responses, viral defense, apoptosis and senescence.	0.1–1 µm	PML, SP100	Mostly protein content, some subsets contain nascent RNA
Nuclear stress bodies	Heat- and chemical- stress-induced condensates that sequester transcription and splicing factors via SatIII ncRNAs to modulate global transcription and splicing.	2–2.5 µm	HSF1, HAP/SAFB	Satellite III (SatIII) ncRNAs

**Table 3:** BMCs in the human cell cytoplasm [45].

BMC	Function	Size (Diameter)	Key Markers	Representative RNAs
P-bodies	Cytoplasmic mRNP granules enriched in mRNA decay factors that store translationally repressed mRNAs and regulate their turnover and recycling, especially across the cell cycle and during stress.	0.2–0.8 µm	DDX6, DCP1A, GW182	Translationally repressed coding mRNAs (including AU-rich transcripts)
Stress granules (SGs)	Transient condensates that sequester stalled pre-initiation complexes and non-translating mRNAs during stress, protecting them from degradation and priming rapid re-entry into translation upon recovery.	0.1–2 µm	G3BP1/2, TIA-1, TIAR	Non-translating mRNAs, poly(A)^+^ RNAs
RNA transport granules (neuronal)	Neuronal RNA granules that mediate long-distance trafficking of translationally repressed mRNAs along neurites and support localized translation at synapses.	0.15–0.6 µm	FMRP, Staufen1/2, IGF2BP1/ZBP1	mRNAs encoding synaptic and cytoskeletal proteins

### 2.1. Multivalency

Multivalent interactions between and within protein and RNA are central drivers of phase separation. The term multivalency refers to the presence of multiple interaction sites (binding motifs or domains) within a molecule that can simultaneously engage in interactions with various other molecules, typically through many low-affinity interaction sites. When valency and local concentration are sufficient, these weak interactions collectively overcome thermal fluctuations, forming droplets of dense phase [5,8,9].

Proteins that participate in condensate formation provide both multivalent interaction modules and a structural framework that shape condensate properties. They are commonly enriched in intrinsically disordered regions (IDR) and prion-like domains (PLDs), RNA-binding domains (RBDs), arginine-rich (RGG/RG) motifs, and other low-complexity sequences, adding molecular specificity and tuning condensate stability and responsiveness to environmental or cellular cues [9].

Multivalency is not limited to proteins. RNA contributes an equally important layer of multivalency. Individual transcripts often contain multiple recognition motifs and structured elements for RNA-binding proteins (RBPs), enabling simultaneous engagement of several protein partners. RNA-RNA base-pairing and higher-order structures further increase valency and connectivity within condensates. As a result, RNA can function both as a scaffold for condensate nucleation and as a modulator of the condensation state or abundance, tuning the threshold of phase separation [11]. Because RNA modifications can selectively create or erase binding sites for reader proteins and alter local RNA structure, they effectively tune the multivalency of RNA–protein interactions, providing cells with a dynamic tool to modulate the number and strength of connections that drive condensate formation and material properties [10].

**Figure 1:**
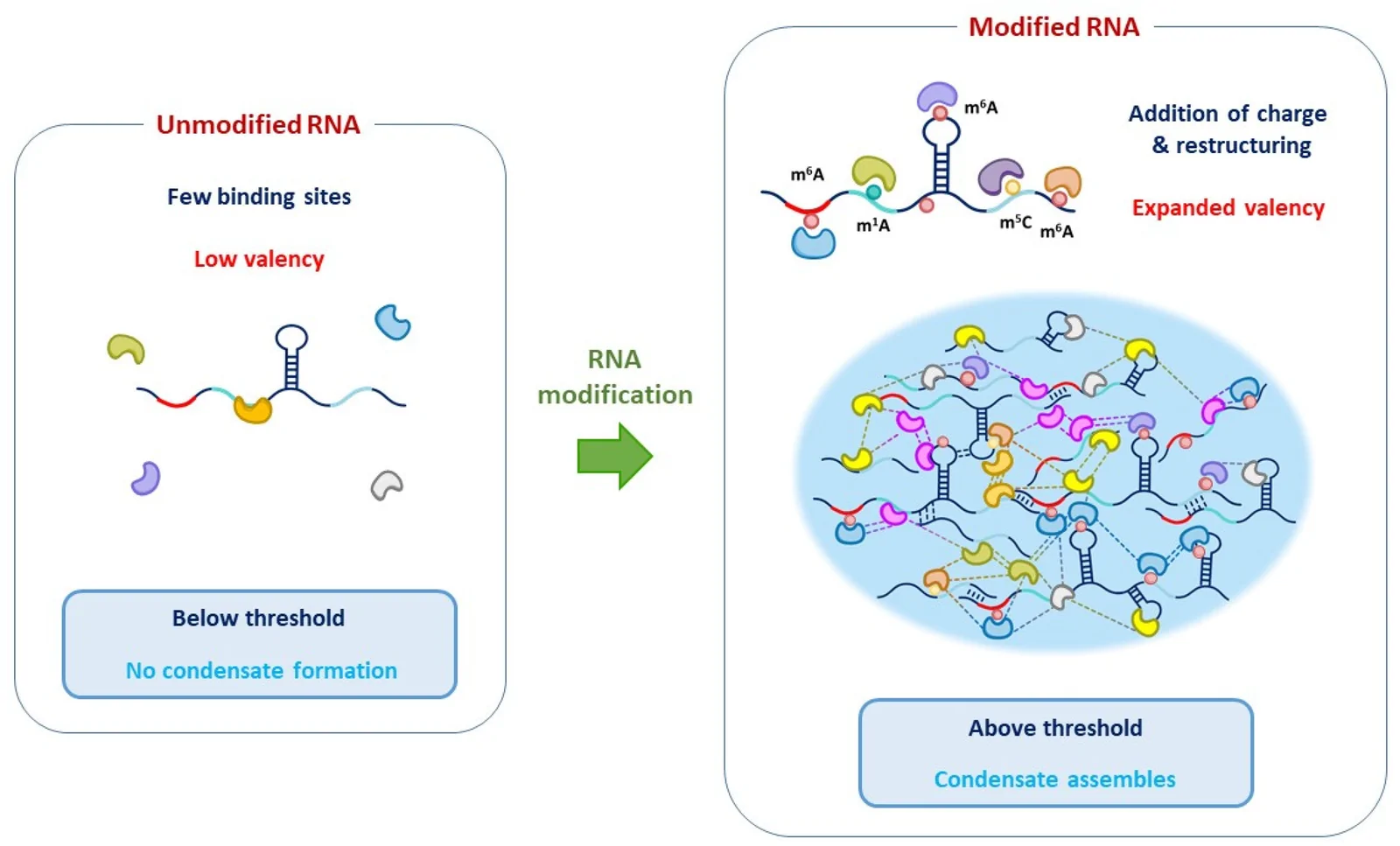
RNA modifications add chemical features (such as charge and structure), creating docking sites for reader proteins, expanding RNA valency and driving multivalent networks that push a transcript above the threshold for condensate assembly. The convoluted interaction networks that promote phase separation, are based on an array of weak protein–protein interactions (dashed lines in the colors of the interacting proteins), RNA–protein interactions (shown by physical proximity) RNA-RNA base-pairing (shown as dark blue base pairing lines).

Although proteins and RNA can each drive phase separation in vitro, the form and function of BMCs in vivo rarely depend on protein or RNA multivalency alone. Instead, phase separation typically emerges from joint highly coordinated networks of protein–protein, protein–RNA, and RNA–RNA interactions, whose combined multivalency dictates condensate formation, composition and dynamics (Figure 1) [10,44,45].

While both proteins and RNA contribute to phase separation, it is important to view their contribution from each perspective, as they contribute distinct layers of selectivity and regulation.

### 2.2. Condensate Formation from a Protein Viewpoint

The capacity of a protein to co-assemble with RNA into condensates is largely encoded in the combination and spatial arrangement of its interaction domains. IDRs, PLDs, RBDs and post-translational modifications (PTMs) together support numerous weak, transient interactions (π–π stacking between aromatic amino acids, cation–π interactions involving arginine/lysine, and electrostatic contacts), allowing proteins to form dynamic, reversible networks that are essential for phase separation [5,46]. The presence and organization of such domains in scaffold proteins such as G3BP, FUS, TDP-43, and hnRNPA1, strongly influence condensate nucleation, composition and dynamics [47,48,49,50,51,52]. The specific contributions of these domains and motifs are outlined below and illustrated in Figure 1.

IDRs and PLDs are ubiquitous in RBPs and they are characterized by the lack of a stable tertiary structure [46]. Their sequence is typically of low complexity, enriched for amino acids that favor flexibility, solubility, weak, transient interactions, and depleted of residues that strongly stabilize fixed three-dimensional structures [48]. IDR sequences often contain recurrent short motifs and blocks of alternating charges [49]. The same composition that disfavors a stable fold favors many weak, transient contacts (electrostatic and cation–π, especially involving Arg/Lys and aromatics), thereby supporting multivalent networks [50].

Interleaved with IDRs are canonical RNA-binding modules such as RNA recognition motifs (RRMs), KH domains, and RGG/RG motifs, which together provide both specificity and multiplicity of RNA contacts. Proteins that carry several RRMs or KH domains, or that combine these structured domains with IDRs, can contact multiple RNAs or multiple sites on the same transcript simultaneously, greatly enhancing multivalency and network connectivity. For example, the RNA-binding protein FUS contains an N-terminal low-complexity domain followed by multiple RGG/RG-rich segments and an RRM, forming an architecture that allows FUS to co-phase-separate with RNA polymerase II and RNA transcripts into nuclear condensates [51,52]. Similarly, the stress-granule nucleator G3BP1 promotes intermolecular RNA-RNA interactions and stabilizes stress-granule assemblies through the combination of folded RNA-binding and dimerization domains with a central IDR [51,53,54].

PLDs are low-complexity regions, also common in many RBPs. They share sequence similarity to prion proteins and are enriched in glutamine and asparagine residues. Like IDRs, PLDs provide multivalent “glue” that supports weak, reversible interactions important for condensation [54,55]. A well-studied example is the TAR DNA-binding protein 43 (TDP-43), a nuclear RNA/DNA binding protein involved in the regulation of RNA processing and stability, whose C-terminal Q/N-rich PLD is required for phase separation [56]. Under stress conditions, TDP-43 can be exported to the cytoplasm and form stress granules (SGs) together with other proteins and RNAs [57].

Whereas many IDRs are enriched in mixed charged and polar residues, classical PLDs show a strong bias for glutamine and asparagine, which predisposes them to more solid-like, aggregation-prone states compared with typical IDRs.

RBDs modulate phase separation of RBPs in diverse and context-dependent manners. Proteins containing PLDs, IDRs, or low complexity sequences frequently also harbor RBDs, suggesting functional cooperation between these modular elements [58]. In some RBPs, the IDRs or PLDs provide the primary driving force for condensate formation, whereas RBDs fine-tune condensate dynamics, composition and material properties, even independently of RNA binding [58]. RNA itself can further regulate condensation by serving as a multivalent scaffold that promotes phase separation, or by altering condensate liquidity through interactions with RRMs or other RBD elements. Importantly, strong specific binding or high RNA-to-protein ratio can inhibit phase separation and instead favor discrete stoichiometric complexes [16,59,60].

RGG/RG motifs represent a prominent example of RBD elements embedded within IDRs, where they can function either as drivers or modulators of phase separation, depending on the protein and RNA context. For example, the C-terminal tail of T-cell intracellular antigen-1 (TIA-1) contains an RGG sequence within an IDR that can serve as a modulator. Binding of Zn^2+^ ions to this segment changes the behavior of TIA-1 with respect to phase separation, shifting the balance between liquid droplets and fibrillar aggregates in SG-like condensates. In this case the RGG/RG region fine-tunes condensate formation and maturation rather than serving as the sole driver [61]. Mixed-charge, low-complexity regions enriched in both acidic and basic residues, as well as arginine-rich motifs (such as RGG/RG boxes) are particularly effective modulators of condensate behavior because they couple strong electrostatic interactions with the RNA backbone to promiscuous protein–protein contacts. These sequence features are recurrent in RGG-motif proteins that partition into SGs, nucleoli, and other RNP condensates [62,63].

Oligomerization domains also contribute to phase separation by promoting self-association into dimers, oligomers, or higher-order assemblies. These domains increase the effective valency of a protein, thus enhancing multivalent intermolecular interactions that drive network formation and condensation. Common oligomerization modules include domains such as coiled-coil, leucine zippers, PB1, SAM, and other structured interaction motifs. By clustering otherwise weak interaction elements, such as IDRs and RBDs, oligomerization domains lower saturation concentration needed for condensate formation and influence their size, dynamics and state properties. However, their effect is context-dependent: oligomerization can promote phase separation when it increases productive multivalency, but stable dilute-phase oligomers may compete with condensate formation under certain conditions [64,65,66].

Protein post-translational modifications (PTMs) are powerful regulators of phase separation because they directly change the charge, hydrophobicity, and interaction valency of condensate-forming proteins. Phosphorylation, acetylation, ubiquitination, arginine methylation, and polyADP-ribosylation promote condensate assembly or trigger dissolution by strengthening or weakening the multivalent contacts that drive phase separation. A well-studied example is arginine methylation in RGG/RG-rich RBPs such as hnRNPA1 (heterogeneous nuclear ribonucleoprotein A1). hnRNPA1 contains two N-terminal RRMs and a C-terminal glycine-rich, prion-like low-complexity domain that are sufficient to drive phase separation, promoting SG assembly. Arginine methylation within the RGG motif region at the C-terminus, reduces positive charge density and prevents close interaction with the phosphate backbone of RNA, thereby fine-tuning the binding strength of hnRNPA1 with its target transcripts [67,68]. Accumulating evidence highlights PTMs as “switches” that tune condensate formation, composition, and material properties in signaling, transcription, and tumor biology (for example [69]).

Together, these protein-encoded features create a tunable “interaction code” that determines when, where and how proteins enter condensates. By adjusting the number, strength and geometry of weak contacts, cells can rapidly remodel condensate formation, composition and material properties in response to changing conditions. Viewed from this protein-centered perspective, phase separation emerges as an actively regulated process rather than a purely passive outcome of macromolecular crowding.

### 2.3. Condensate Formation from an RNA Viewpoint

Analogously, RNA itself is a modular, multivalent determinant of condensate behavior. Repeated sequence motifs, structured elements such as stem-and-loops or G-quadruplexes, and clusters of epitranscriptomic marks together create multiple, spatially organized binding sites for proteins and other RNAs. By varying motif copy number, structure, and modification density, cells can tune the valency and geometry of RNA-protein and RNA-RNA interaction networks, thus influencing which condensates form, which transcripts are recruited, and how their material properties evolve. Thus, RNA contributes a layer of multivalency, by acting as a critical scaffold whose abundance, length, structure, and modification landscapes collectively set the threshold for phase separation (Figure 1) [14].

RNA length is a primary factor of RNA ability to act as a scaffold within condensates. Longer transcripts can simultaneously engage multiple RBPs and other RNAs, increasing interaction valency and lowering the concentration threshold for condensation. Conceptually, a long RNA can consist of a higher number of binding motifs and complementary stretches, producing a more convoluted structure, which can act as a flexible hub to promote interactions with many partners. Whereas, a short RNA is limited in its structuring and number of motifs, reducing the amounts of proteins and RNAs it can interact with, and, therefore, not efficient in seeding phase separation. RNA length is an important determinant of SG partitioning [70]. SGs are preferentially enriched for long transcripts, likely because they provide more sites for weak RNA-RNA and RNA–-protein interactions [71]. Longer transcripts also tend to contain long internal exons, which are preferentially m^6^A-modified [72]. Although m^6^A is not universally required for SG formation or mRNA partitioning [21], several studies support a role for the m^6^A-YTHDF axis in promoting granule assembly [18]. The magnitude of this contribution remains debated, with some studies attributing the preferential enrichment of long transcripts largely to m^6^A [72], whereas others find that transcript length and translational status are the dominant determinants and that m^6^A contributes only modestly [21].

Altering RNA length by alternative splicing or transcriptional termination can redirect condensate localization. For example, the long noncoding RNA (lncRNA) NEAT1 has two isoforms, generated by alternative 3′-end processing: its short isoform does not scaffold paraspeckles (PS), while its long isoform, NEAT1_2, acts as the primary architectural PS scaffold [73,74,75]. Another example is the lncRNA MALAT1 that is processed into at least two splice isoforms of different lengths and behaviors: mapping of cis-acting localization regions using engineered fragments and deletion constructs revealed that specific sequence- and structural-elements are required for nuclear speckle association, rather than a simple long-versus-short splice-isoform dichotomy [76]. The long MALAT1 variant contains multiple binding motifs for splicing factors and other RBPs, enabling it to act as a multivalent scaffold at the periphery of nuclear speckles (NSs), whereas the short splice variant lacks these interaction surfaces and does not contribute to condensate formation [76].

RNA structure is a key determinant of how transcripts participate in condensate formation because it alters the exposure and spatial arrangement of binding sites. RNA secondary and tertiary structures, including stem-and-loops, bulges, and G-quadruplexes can create high-affinity docking surfaces for specific proteins [77,78,79,80] and increase intermolecular connectivity, thereby promoting RNA condensation or RNA-RNA base pairing between different transcripts, increasing network connectivity [81,82]. On the other hand, long, unstructured sequences form pervasive intermolecular interaction networks that support mesh-like condensation [83]. Thus, both structured motifs and extended unstructured regions can promote condensation through distinct interaction mechanisms, allowing RNA architecture to tune condensate assembly, morphology, and material properties.

RNA restructuring alters the shielding and exposure of different binding motifs, effectively changing transcripts’ valency and enabling crosstalk between multiple partners. Depending on the specific structural rearrangement and molecular context, these changes can influence condensate formation and stability [81,82]. Members of the conserved DEAD-box family of ATP-dependent RNA helicases are important regulators of the assembly and turnover of RNA-containing condensates. For several DEAD-box proteins, the ATP-bound, RNA-associated state promotes RNA retention and condensate assembly, whereas ATP hydrolysis facilitates RNA release and condensate turnover [84,85]. However, the effects of DEAD-box proteins are context-dependent. The translation-initiation factor eIF4A acts as an ATP-dependent RNA chaperone that limits intermolecular RNA–RNA interactions, thereby reducing RNA condensation in vitro and restricting stress-granule formation in cells [59]. More broadly, DEAD-box helicases remodel RNA and RNP complexes by disrupting or rearranging RNA–RNA and RNA–protein interactions, and some family members can resolve structured RNA elements, including G-quadruplexes [78]. Their influence on condensates may therefore extend beyond direct effects on assembly and dissolution to the regulation of RNA structure and network connectivity. RNA structures also create high-affinity docking platforms, increasing RNA-RNA networks. Thus, RNA structures can dictate which proteins are recruited and how the resulting network is spatially organized. A well-studied example is disease-linked G4C2 hexanucleotide repeat RNAs. In familial amyotrophic lateral sclerosis (ALS) and frontotemporal dementia (FTD), these G-quadruplexes-forming transcripts undergo protein-independent phase separation forming nuclear foci [81]. The content and clustering of certain recognition elements is also critical for condensate sequestration. For instance, AU-rich motifs are associated with poor translation efficiency, correlating with P-body localization [86].

Valency is a key determinant of RNA-driven phase separation. The ability to generate multivalent interactions either with proteins or RNA base-pairing is an important determinant that correlates with phase transition. Increasing valency shifts the phase boundary so that even low RNA concentration suffices to nucleate droplets [87,88,89]. For example, transcripts consisting of more than 30 CAG triplet repeats are retained in NSs [81]. In contrast, RNAs with few motifs require a much higher concentration to condense [90]. This effect depends on intrinsic RNA sequences, or RNA structure, affecting the ability to bind dedicated proteins. In essence, each additional binding site acts like an extra “sticker” in the multivalent network [90].

Proteins can augment valency by serving as an “RNA condensers,” promoting intermolecular RNA-RNA interactions, effectively increasing network connectivity, as in the case of the SG protein G3BP1 [91].

RNA modifications: BMC formation is driven by multivalent interaction networks. Proteins partially dictate where and how condensates form and what material properties they display (see Section 2.2). In parallel, RNAs provide a matching layer of control, affecting which condensates nucleate, which proteins are recruited, and how sensitively these assemblies respond to cellular cues. Similar to PTMs, RNA modifications can regulate phase separation by introducing new chemical features including changes in charge, structure and hydrophobicity, altering interaction networks and effectively expanding valency, for example by creating additional binding sites for reader proteins [10]. However, the relationship between modification number and condensate behavior is not necessarily linear and depends on transcript context, reader availability, and cellular state Phase separation plays a role in regulation of RNA modification processing, warranting their metabolism. Condensation itself can influence RNA modification pathways due to local concentration of writers, erasers, and readers with their mRNA substrates within the condensate. For example, *N*^6^-methyladenosine (m^6^A), enhances phase separation of polymethylated transcripts in vitro and in cells [19]. These transcripts form multivalent scaffolds that are bound by cytosolic members of the YTH domain containing family, YTHDF1-3 [19].

Together, these parameters underline that biomolecular condensates and RNA modifications are not independent layers of regulation, but parts of an integrated system in which RNA acts simultaneously as scaffold and substrate, and in which phase separation both interprets and reshapes the epitranscriptomic landscape. The bidirectional relationship between phase separation and the RNA modifying processes will be discussed in the following section.

## 3. RNA Modifications Landscape Affect Phase Separation

RNA modifications add an additional dimension to the process of phase separation by effectively changing the valency and the interaction profile of transcripts. This raises a central question: how can chemically modified transcripts promote or prevent binding of RBPs? The answer depends on the nature of each modification. Chemical adducts within an RNA sequences can be read directly by dedicated protein domains that recognize the modified sequence, such as YTHDFs (see below) or act indirectly by inducing RNA refolding of local structures, exposing or occluding sequence motifs for proteins to bind, such as hnRNPs (see below). In this way, RNA modifications tune the interaction landscape between RNAs and RBPs, thereby modulating the formation and properties of BMCs such as stress granules, P-bodies, and nuclear bodies [10].

Various RNA modifications are linked to phase separation, and readers of these epitranscriptomic marks are involved in forming condensates. In the following sections, these individual RNA modifications are discussed, and experimental evidence that link each mark and its readers to condensate formation are presented.

### 3.1. N^6^-Methyladenosine (m^6^A) Regulates Phase Separation

m^6^A is the most studied dynamic mRNA modification. It is regulated by its writers, erasers and a growing list of reader proteins, including member of three protein families: YTH-domain containing proteins (YTHDFs and YTHDCs), insulin-like growth factor-2 mRNA-binding proteins (IGF2BPs), and heterogeneous nuclear ribonucleoproteins hnRNPs [26,29,92]. Studies over the last decade revealed that m^6^A is deeply intertwined with BMC formation by increasing the valency of RNAs and recruiting reader proteins, consequently stimulating droplet formation [93]. Depending on cellular context, each m^6^A residue increases the valency of the transcript, thus, multiple m^6^A groups (termed poly- or hyper-methylation) lower the concentration threshold needed for condensation [19,20].

YTHDFs: The m^6^A cytoplasmic reader proteins, YTHDF1-3, contain an N-terminal IDR/low-complexity domain and a conserved YTH domain at the C-terminus. The YTH domain recognizes m^6^A through a hydrophobic “aromatic cage”, which tethers YTHDFs to m^6^A-modified transcripts. In contrast, the N-terminal low-complexity domain does not bind m^6^A directly but forms multivalent protein–protein interactions. Poly-methylated transcripts recruit many YTHDFs, juxtaposing their low complexity domains, forming mRNA condensates that can form BMCs such as P-bodies and SGs [13,19,20].

Importantly, the isolated YTH domain, despite robust m^6^A recognition, does not undergo condensation on its own, underscoring that m^6^A binding and phase separation rely on distinct structural elements [19]. Although all three YTHDF paralogs bind m^6^A sites with similar affinity, they exhibit distinct condensate behaviors, reflecting divergence in their N-terminal low-complexity domains and partners. YTHDF1 and YTHDF2 have dissimilar low-complexity domains that show different propensities and dynamics of condensate formation in vitro and in cells. Functionally, YTHDF1 is more linked to translational control, such as SGs, whereas YTHDF2 is more associated with RNA decay and is rich in P-bodies [18,20]. However, these differences are context-specific, since the mRNA targets of YTHDF1-3 often overlap and they act cooperatively [20].

Several observations highlight the role of m^6^A-YTHDF interaction in phase separation in cells: Reducing mRNA m^6^A levels by depletion of its writer, METTL14, decreased YTHDF2 recruitment to P-bodies under normal conditions [19]. A dominant negative YTHDF1 variant in which its low-complexity domain was replaced with CRY2olig domain (that oligomerizes undergo blue light) partially decreased SG formation [18], and mutations in the YTH domain of YTHDF2 reduced its enrichment in SGs under stress [19]. Moreover, depletion of YTHDF1 and/or YTHDF3 impaired SG formation. Together with domain-swap and YTH-mutant experiments, these findings support a model in which m^6^A recognition by the YTH domain licenses transcript selection, while the N-terminal low-complexity region provides the multivalent contacts needed for condensate assembly [18,19]. Additional regulation is provided by PTMs of YTHDFs. O-GlcNAcylation of YTHDF1 and YTHDF3, but not YTHDF2, affecting condensation, stability and dissolution of SGs, and enabling better recovery from stress [94].

Still, it is important to distinguish the effects on bulk SG assembly from the partitioning behavior of individual endogenous transcripts. Harnessing engineered m^6^A-modified reporter mRNAs and hyper-methylated transcripts, revealed that elevated m^6^A methylation can promote recruitment of YTHDFs and enhance SG assembly in cells and in droplet formation, at least in the specific experimental contexts tested, [15,18,95]. A spatiotemporal live cell imaging system enabled monitoring m^6^A accumulation in SGs [96] and showed that YTHDF2 regulates SG stability through interaction with G3BP1 in an m^6^A-dependent manner [96], further enhancing a role for m^6^A reader proteins in condensate assembly. In contrast, single-transcript analyses revealed only a limited effect of global m^6^A decrease on SG assembly, based on a set of endogenous mRNAs, suggesting that m^6^A contributes only modestly to this process [21].

Taken together, these observations draw a complex, context-dependent picture: divergent low-complexity domains provide the three YTHDF paralogs with distinct phase-separation behaviors and partner repertoires, forming different condensates, even though they can all bind the same m^6^A sites.

YTHDC1: Similar to YTHDFs, YTHDC1 also harbors a YTH-domain. It is a multifaceted nuclear protein that plays a role in a wide range of regulatory mechanisms in gene expression [97]. Its ability to form BMCs is tightly coupled to its functions in transcriptional regulation, RNA processing, and nuclear export. YTHDC1 binds m^6^A-modified mRNAs and undergoes phase separation to form dynamic nuclear bodies [22]. These nuclear YTHDC1-m^6^A condensates (nYACs) appear in larger numbers in acute myeloid leukemia (AML) and are enriched in leukemogenic transcripts and associated factors. nYACs maintain cell survival and the undifferentiated state of AML, making them critical for leukemia maintenance [22]. Mechanistically, these BMCs are essential for the ability of YTHDC1 to protect m^6^A-modified transcripts from degradation by the polyA tail exosome targeting (PAXT) complex [22].

YTHDC1 also forms BRD4 transcriptional condensates at the loci of active enhancers by binding m^6^A-modified enhancer RNAs (eRNAs). YTHDC1-methylated-eRNA condensates activate transcription by clustering transcriptional co-regulators and RNA processing machinery [23]. Chromatin-bound YTHDC1 is also implicated in recognition of METTL3- and METTL16-m^6^A marks on chromatin-associated RNAs (carRNAs) [98]. A recent study demonstrated that YTHDC1-METTL16-dependent m^6^A caRNA condensation recruits splicing factors to transcription complexes, forming BMCs that couple transcription with co-transcriptional splicing [98,99]. The initial work on m^6^A and caRNAs (including promoter-associated RNAs, enhancer RNAs and repeats) in mouse embryonic stem cells showed that YTHDC1 binds a subset of these m^6^A-modified caRNAs and promotes their decay via the NEXT complex, thereby reducing caRNA abundance, but it is not clear if the recruited NEXT complex is associated with phase separation [99].

In pulmonary fibrosis models, YTHDC1 binds an m^6^A-modified pro-fibrotic lncRNA (lnc668) and undergoes phase separation to assemble a nuclear export module with SRSF3, ALYREF, and XPO5 at nuclear pores. These YTHDC1 condensates promote nuclear export of m^6^A-modified lnc668 to enhance its cytoplasmic functions in driving fibroblast-to-myofibroblast differentiation. Disruption of YTHDC1 phase separation impairs lnc668 export from nucleus to cytoplasm and fibrotic progression [100].

IGF2BPs: IGF2BP1-3 are a conserved family of cytoplasmic m^6^A readers that have been directly linked to phase separation. All three IGF2BPs share a similar modular organization, in both order and spacing of domains, consistent with similar biochemical functions [26,101]. This common architecture consists of two N-terminal RRMs followed by four tandem C-terminal K-homology (KH) domains, arranged as two tandem di-domains (KH-2 and KH3-4). The KH domains form the principle m^6^A recognition module, creating a cleft that preferentially recognizes GG-m^6^A-C motifs and stabilizes associated target mRNAs by protecting them from degradation [26,102]. Deleting or mutating the KH domains abolishes m^6^A-dependent binding, whereas the RRMs contribute to general RNA binding but are insufficient for selective m^6^A recognition [26].

IGF2BP1, for instance, acts a phase-separating m^6^A reader whose KH3-KH4 di-domain and flanking low complexity linker sequences drive condensation [26]. PTMs of IGF2BP1 within the disordered linkers modulate condensate material properties, altering condensate size, number, and internal dynamics without eliminating RNA binding, indicating that the protein can be “tuned” between more or less cohesive states [27].

In cells under osmotic or oxidative stress, IGF2BP1 self-clusters within seconds, preceding recruitment of SG markers such as G3BP1 and TIA1, supporting a role for IGF2BP1 as an early scaffold and nucleator of stress-related BMCs [28,103]. Within these condensates, IGF2BP1 binds m^6^A-modified RNAs and enhances the stability of selected targets protecting them from degradation while they are sequestered in granules [26,101]. However, experiments that inhibit visible SG formation while preserving IGF2BP1-mRNA interactions show that SGs are dispensable for IGF2BP1-mediated mRNA stabilization, indicating that an explicitly m^6^A-dependent “condensate-protection” mechanism for all targets is supported but is not a general rule. More broadly, IGF2BPs act as stabilizing m^6^A readers and organize multivalent mRNP condensates, supporting their role as m^6^A-dependent “stabilizing hubs” [26], but the extent to which their phase separation behavior is required for each regulatory outcome remains an active area of investigation.

m^6^A switch: The original “m^6^A switch” concept emerged from studying hnRNPC and hnRNPG, which bind their transcript targets in an m^6^A-dependent manner even though they do not recognize the methylated sequences itself [29,30]. In such cases, m^6^A can destabilize local RNA structures and promotes an RNA conformation transition, exposing otherwise buried recognition motifs, to recruit RBPs such as hnRNPs [15]. Beyond altering RBP access, m^6^A switch can also regulate the kinetics of RNA-RNA interactions, so that modification at specific sites have the potential to rewire interactions with other transcripts, and tuning multivalent interaction dynamics [29,30]. m^6^A also shapes nuclear condensates beyond the reader-driven examples above. In PSs, m^6^A destabilizes the scaffolding lncRNA NEAT1, so that its removal by the m^6^A eraser ALKBH5, which itself phase-separates through its C-terminal IDR, stabilizes NEAT1 and promotes hypoxia-induced PS assembly [104]. Conversely, nuclear stress bodies (nSBs) act as sinks for the m^6^A machinery: during stress the m^6^A writer complex ac-cumulates in nSBs and methylates the architectural HSATIII lncRNA, which sequesters the nuclear reader YTHDC1, lowers the nucleoplasmic availability of m^6^A-associated factors and thereby suppresses m^6^A-dependent splicing of other pre-mRNAs [105]. These cases show that m^6^A can control condensate assembly both by tuning scaffold-RNA stability and by partitioning the modification machinery itself.

Although the term “m^6^A switch” originally refers to the structural shift described above, m^6^A can also serve as a biophysical valency switch. Increasing the number of m^6^A sites on a single transcript can enhance its scaffold capacity by enabling simultaneous recruitment of multiple readers such as YTHDFs (see above) [19]. In such cases, the “switch” is less about reshaping the RNA and more about changing condensate propensity by altering how many readers and co-factors an RNA can simultaneously engage [15].

A third, functionally distinct m^6^A-dependent switch has been described for maternal FMR1 granules in Drosophila [106]. During early embryonic development, FMR1 forms RNA-protein granules that store large pools of maternal mRNAs, a subset of which is hyper-m^6^A methylated, and preferentially bound by FMR1. Consequently, when FMR1 binds hyper methylated transcripts, those mRNAs act as multivalent scaffolds that drive FMR1 granules into a more dynamic, liquid-like state that interacts differently with the decay machinery. Thus, m^6^A acts not only as a passive mark but it also dictates which mRNAs are loaded to FMR1 granules, regulating maternal RNA clearance, which is essential for the maternal-to-zygotic transition [106]. The study demonstrated that a subset of m^6^A-modified maternal mRNAs FMR1-RNP granules undergo a dynamic phase switch, changing their material state and thereby controlling maternal RNA clearance [106].

The m^6^A switch concept illustrates how altering m^6^A deposition can transition RNAs between non-condensing and condensing states, changing which readers bind it and how strongly they multimerize.

### 3.2. Phase Separation and N^1^-Methyladenosine (m^1^A) mRNA Modification

While m^6^A is highly prevalent and best-characterized mRNA modification in the context of BMCs, accumulating information link another epitranscriptomic mark, m^1^A, to condensate formation [107].

Internal m^1^A in cytosolic mRNA has been the subject of considerable debate: Several studies demonstrated a large number of m^1^A site in human and mouse mRNA [108,109]. However, a single-nucleotide mapping study in human cells, identified only a handful of internal m^1^A sites in cytosolic mRNAs arguing that they are rare and typically present at low stoichiometry. Moreover, the validated sites in this study were introduced by the TRMT6/TRMT61A complex in tRNA T-loop-like structures [110]. Subsequent re-evaluation of earlier maps revealed many 5′UTR m^1^A sites were identified due to antibody cross-reactivity [111]. However, differences in mapping strategies and analysis criteria are probably the reason for the divergent estimations of m^1^A site numbers and stoichiometry [112]. Within this framework, several examples present specific mechanistic cases on selected transcripts.

m^1^A carries a positive charge at physiological conditions and it disrupts Watson–Crick A-U base-pairing, strongly affecting local RNA structure and interaction surfaces [113], which in principle alters how RNAs engage in multivalent networks within condensates. In contrast to m^6^A, where dedicated reader proteins and reader-driven phase separation are well-characterized, the mechanistic details of how m^1^A regulates phase separation are less clear.

TRMT6/61A: Under stress, such as heat shock, m^1^A appears to influence phase separation primarily by marking which mRNAs enter SGs and how well they are protected there, rather than by acting through a well-defined reader system [31]. During heat stress, the m^1^A writer complex TRMT6/61A, which deposits internal m^1^A in cytoplasmic mRNAs, relocalizes to SGs, resulting in enrichment of m^1^A in SG-associated RNAs compared to the bulk transcriptome [31]. Introducing a single TRMT6/61A-dependent m^1^A motif to a reporter mRNA promotes its sequestration into SGs during heat shock and enhances recovery of its translation, once stress is relieved, consistent with the concept that m^1^A-tagged transcripts are preferentially routed into SG condensates where they are shielded from degradation and released to the cytoplasm for translation after stress is resolved [31].

These examples show that while the global abundance of m^1^A remains to be determined, this epitranscriptomic mark is clearly present and functional in multiple sites.

TDP-43: Recent structural and biochemical work identifies TDP-43 as an m^1^A reader [32]. TDP-43 contains an N-terminus oligomerization domain [114], two RRM domains and a C-terminal low complexity, glycine-rich domain that drives phase separation [115]. In the CNS, TDP-43 can interact with SMN, serving as a scaffold for nuclear bodies [116]. From a condensate perspective: the RRMs provide RNA multivalency, the N-terminal domain organizes oligomers, and the low complexity domain sets the condensate behavior, switching between reversible liquid droplets and irreversible amyloid-like aggregates in disease [115]. TDP-43 is present in multiple nuclear BMCs [117], however its m^1^A-dependent role in phase separation was only recently uncovered.

ALKBH3: The m^1^A eraser, ALKBH3, demethylates m^1^A from mRNA. Recent direct experimental evidence link m^1^A to the formation of PML nuclear bodies through regulation of the m^1^A-methylated state of SP100A transcripts, which encode a core structural component of these bodies [33]. In normal melanocytes, histone lactylation upregulates the m^1^A eraser, ALKBH3, leading to hypo-methylation of SP100A transcripts. The m^6^A reader YTHDF1, which reads both m^6^A and m^1^A, preferentially binds m^1^A-methylated SP100A mRNAs, increasing their stability and expression, and in this manner promoting the formation of tumor-suppressive PML nuclear condensates [33]. In ocular melanoma, ALKBH3 is aberrantly upregulated resulting in reduced m^1^A-methylation of SP100A mRNAs, which in turn, reduces YTHDF1 binding of these transcripts, consequently attenuating PML body formation [33]. Functionally, loss of PML condensates correlates with enhanced tumor progression, and silencing ALKBH3 restores PML bodies and suppresses melanoma growth in vitro and in vivo. Depletion of SP100A expression reverses this effect [33].

It should be noted that direct evidence for YTHDF1 acting as an m^1^A-dependent driver of phase separation currently rests on this SP100A example; whether YTHDF1 functions as a more general m^1^A-controlled regulator of condensate formation beyond this context will require further direct binding and cellular validation in additional systems.

Together, these examples suggest that although mechanistic details are still emerging, m^1^A can influence both the composition and abundance of specific nuclear and cytoplasmic condensates by modulating RNA structure, RNA-protein recognition, and the expression of key scaffold proteins.

### 3.3. Phase Separation Is Linked to 5-Methylcytidine (m^5^C) mRNA Modification

m^5^C is also experimentally linked to phase separation through its readers belonging to the Y-box protein family (YBX1 and YBX2, see below). Other m^5^C readers such as ALYREF in nuclear export, have not yet been mechanistically linked to phase separation [38,118]. These proteins share basic architectural features that enable m^5^C-linked condensation: a central cold-shock domain that provides specific RNA recognition, flanked by IDRs that supply the multivalent interaction network needed for phase separation [37,119,120,121]. The cold-shock domain binds m^5^C-modified RNA sequences, whereas the low-complexity IDRs, which are enriched in basic and aromatic residues, support weak, multivalent protein–protein and protein-RNA contacts that allow YBX condensates to assemble and remodel in response to changes in m^5^C-marked RNA cargo [37,119,120,121].

YBX1 has been shown to undergo phase separation in vitro and in cells, forming condensates that partition into cytoplasmic mRNA-protein (mRNP) granules, and P-body-like compartments, along with selected miRNAs, such as miR-223, before these are sorted into exosomes [34]. Mutations in its low-complexity IDRs that suppress phase separation also reduce YBX1 incorporation into cellular condensates and impair its function in miRNA sorting, indicating that condensation is functionally relevant in cells [34]. While there is solid evidence that YBX1 is an mRNA m^5^C reader, it remains unclear whether its miRNA-sorting function is mediated by m^5^C. Studies show that binding to m^5^C-modified mRNAs can lead to phase separation-dependent transcript stabilization. For example, YBX1 directly binds m^5^C sites in the 3′UTRs of COX5B mRNAs, enhance their stability and expression [36]. Similarly, YBX1 recognizes m^5^C sites in the 3′UTR of RNF115 mRNAs [35].

YBX2 provides a clear example of how an m^5^C reader can drive phase separation. In vitro, YBX2 undergoes phase separation, and the presence of m^5^C-containing RNAs markedly lowers the concentration threshold for droplet formation, and increases droplet size and stability. Mutation of a key aromatic residue in the m^5^C-binding pocket disrupts both preferential m^5^C binding and phase separation, demonstrating that m^5^C recognition and condensation are structurally coupled [37,122]. In cells, YBX2 forms cytoplasmic mRNP granules whose assembly depends on its RNA-binding activity and is enhanced by m^5^C-modified transcripts, consistent with YBX2 acting as an m^5^C-dependent scaffold that organizes stable cytoplasmic condensates for selected mRNAs [37,121].

Together, these findings support a model in which m^5^C marks select transcripts that are preferentially loaded into YBX-driven condensates, which then act as stabilizing hubs that tune mRNA fate within the cytoplasm.

### 3.4. Phase Separation Is Linked to Other RNA Modifications

Database-scale analyses show that diverse post-transcriptional events, including alternative splicing, alternative polyadenylation, A-to-I editing, and multiple RNA modifications, are all represented among RNAs enriched in BMCs, underscoring that many layers of RNA regulation feed into condensate association [5,40,41]. The link of A-to-I editing, Ψ and m^7^G to phase separation is mostly conceptual and indirect. Still, they are all known to impact RNA structure and recognition by proteins [5,40,41].

A-to-I RNA editing converts adenosine to inosine in double-stranded RNA, changing base-pairing from A=U to I≡C, altering local duplex stability and recognition by RBPs. However, direct experiments, where edited in contrast to unedited RNAs affect phase separation are still lacking. Most statements are extrapolations from known structural and interaction effects rather than biophysical condensation assays. In parallel, a rapidly expanding body of evidence links ADAR1- and ADAR2-mediated A-to-I editing to stress responses and innate immune regulation in multiple tissues [42], but whether these enzymes or their edited transcripts directly drive or modulate BMC formation remains to be determined.

Pseudouridine (Ψ) is a highly abundant RNA modification. It improves base stacking and can contribute to stabilization of local RNA structures while altering RBP recognition. Thus, Ψ is expected to tune BMC formation and dynamics trough RNA restructuring [5].

m^7^G at the 5′ cap of mRNA as well as in internal positions alters charge distribution and stacking [43]. m^7^G-capped mRNAs are preferentially engaged by cap-binding complexes and translation factors that themselves participate in condensation, where m^7^G together with m^6^A likely influence condensate composition and dynamics [5]. Internal m^7^G in mRNAs and miRNAs has been linked to changes in structure and translation, but, again, direct experiments linking them to phase separation are still unavailable and current links are mainly based on studies revealing how m^7^G changes binding to cap-binding proteins and translation machinery that reside in BMCs [43].

Beyond the modifications discussed above, other RNA modifications may also modulate condensate behavior by altering RNA structure and protein binding, but direct mechanistic evidence connecting these marks to phase separation remains limited and largely inferred from their known effects on RNA chemistry and RNP assembly [5]. Table 3 summarizes the available data on RNA modifications linked to BMCs.

## 4. RNA Modifications Affect BMCs in Health, Stress and Disease

RNA modifications influence all main principles of phase separation, from changing RNA valency to rewiring protein–RNA, RNA-RNA and even protein–protein interaction networks within condensates. However, their main contribution is through their impact on RNA metabolism and structure, affecting translation, storage, stability and decay.

Phase separation-generated condensates such as the nucleolus, chromatin-associated hubs, transcriptional condensates at super-enhancers, and developmental RNP granules (for example FXR1 granules) are essential for normal ribosome biogenesis, gene regulation, and germ cell development, illustrating that BMCs are fundamental, physiological organizers of cellular biochemistry rather than phenomena restricted to stress or disease. The role of RNA modifications in stress However, when RNA modifications or their associated writers, readers and erasers become dysregulated, the resulting changes in condensate formation, composition and material properties can contribute directly to disease states.

### 4.1. Phase Separation Under Stress

Cells dynamically remodel mRNA modifications in response to diverse stress conditions, using these marks to re-route transcripts between translation, storage, and decay. Under oxidative stress, heat shock, hypoxia, and nutrient deprivation, many transcripts acquire additional epitranscriptomic marks [108,123,124,125]. Stress-induced epitranscriptomic marks help triage mRNAs into SGs and other RNA-protein condensates, biasing them away from active translation and toward temporary storage or controlled decay [27,31,107]. As described in Section 3.1, the interplay between m^6^A and YTHDFs appears to be context dependent. While the m^6^A-YTHDFs interplay appeared to have a major effect on SG formation and stability in some systems [15,18,95,96], a different study demonstrated that a global decrease in m^6^A had only a minor effect on another system [21]. These studies support a model in which m^6^A-YTHDF interactions can facilitate SG assembly and affect which transcripts are recruited into them, as demonstrated using engineered or hyper-methylated reporters [15,18,95]. At the same time, single-transcript analyses of a set of endogenous mRNAs demonstrated that the absence of m^6^A had only a limited effect on SG partitioning, indicating that other sequence features, RNA structures and protein interactions also play roles in defining SG composition [21]. Taken together, m^6^A is best viewed as a modulator of granule behavior rather than a universal “address label” for all stress-responsive mRNAs. The balance between paralog-specific and cooperative YTHDF functions remains an active area of investigation. A disease-specific illustration of this context dependence, in which lowering m^6^A normalizes pathological SG behavior, is discussed in Section 4.3.1. Other mRNA modifications affect transcript enrichment in SGs under different conditions. During heat shock TRMT6/61A relocalizes to SGs where m^1^A-marked transcripts are preferentially granulated and protected from degradation [31,126]. Similarly, m^5^C and Ψ can change under stress conditions such as nutrient stress or viral infection, altering RNA structure and translation in ways that indirectly influence which mRNAs are available for recruitment into stress-responsive condensates. For example, m^5^C landscape changes during viral infection or under nutrient stress [125].

Overall, stress-regulated mRNA modifications provide cells with a rapid, reversible way to rewire the epitranscriptomic landscape so that transcripts are partitioned into appropriate condensates. This reshapes expression programs, adapting them to the prevailing conditions.

### 4.2. Condensate Rewiring by RNA Modifications in Cancer

In cancer, dysregulated RNA modification pathways often converge on BMCs affecting proliferation, differentiation, survival and stress adaptation. Aberrant expression of writers, erasers and readers change which transcripts are modified and alter the phase behavior of the reader proteins themselves, rewiring condensate composition and function.

#### 4.2.1. Nuclear m^6^A Readers: YTHDC1 Condensates

A prominent example is m^6^A-dependent nYAC formation by YTHDC1 in AML [22]. nYACs are enriched for leukemogenic transcripts and associated factors, protecting them from PAXT-mediated degradation, and in this manner rendering them essential for leukemia maintenance and resistance to differentiation. Mutations in the YTH or IDR domains of YTHDC1 disrupt nYACs condensation and reduce the stability of m^6^A-modified YTHDC1 target transcripts, decreasing AML cell survival [22]. Thus, the m^6^A-YTHDC1-nYAC axis illustrates how m^6^A-associated condensates can act as an oncogenic hub.

YTHDC1 is also implicated in phase separation of eRNAs and caRNAs. The direct mechanistic study in an MCF7 breast cancer cell model showed that m^6^A-modified eRNAs, YTHDC1, and BRD4 condensates are strong associated with transcription enhancer activation and gene expression [23]. In these condensates, loss of YTHDC1 reduced phase separation, robustly inhibited transcription of eRNAs but did not affect their stability, suggesting a mechanism different than that of nYACs, in which m^6^A deposition at eRNAs can reshape enhancer condensates in ways that favor malignant programs [23]. These findings support a model where m^6^A-marked eRNAs recruit YTHDC1 into enhancer-localized condensates, reinforcing BRD4 and other co-activators and thereby amplifying transcription of cancer-relevant genes [23].

In mESCs, YTHDC1 binds m^6^A-modified retrotransposon-derived RNAs (IAP, ERVK, LINE1), which tether YTHDC1 to their chromatin loci and allow it to recruit SETDB1 and other chromatin modifiers [127]. Mechanistically, m^6^A-marked retrotransposon RNAs act as scaffolds that recruit YTHDC1 and SETDB1 to chromatin, supporting H3K9me3 heterochromatin and suppressing transcription of these elements [127]. YTHDC1 depletion decreases SETDB1-mediated H3K9me3 heterochromatin and reactivates the silenced retrotransposons [127].

Focusing on triple-negative breast cancer (TNBC) revealed that YTHDC1 promotes m^6^A-dependent upregulation of FOXM1, which in turn elevates CENPA expression and enhances glycolysis and tumor progression [128]. Depletion of m^6^A reduces YTHDC1-dependent FOXM1 expression, suggesting a YTHDC1-m^6^A-FOXM1-CENPA axis that drives TNBC growth and metabolic reprogramming [128]. Although this study couples nuclear m^6^A recognition by YTHDC1 to oncogenic transcriptional programs, a direct link to phase separation was not addressed. However, FOXM1 and BMCs are linked in different systems [128,129,130], suggesting that FOXM1 can participate in nuclear bodies or transcriptional hubs under certain stimuli in other systems [22].

In ovarian cancer, YTHDC1can act as a tumor suppressor through a PIK3R1-STAT3-GANAB axis [131]. In this context, YTHDC1 stabilizes m^6^A-modified PIK3R1 transcripts and increases PIK3R1 expression, which suppresses GANAB, perturbs N-glycan biosynthesis and restrains malignant behavior [131]. Among the components of this pathway, only STAT3 has been experimentally shown to undergo phase separation and form condensates in response to stimulation in other cellular systems [132], whereas PIK3R1 and GANAB have not yet been directly linked to biomolecular phase separation.

In advanced-stage ovarian cancer, YTHDC1 functions as a pro-tumorigenic factor, helping cancer cells escape senescence by stabilizing m^6^A-modified TERT mRNA [133]. TERT mRNA has not yet been directly implicated as a scaffold or client in BMCs, but this example underscores that YTHDC1 can either suppress or promote tumor progression depending on its target RNAs and the engaged pathways.

#### 4.2.2. Cytoplasmic m^6^A Readers: YTHDF and IGF2BP Condensates

Beyond nuclear m^6^A readers, cytoplasmic m^6^A readers and other modifications further diversify how RNA marks rewire condensates in cancer. YTHDFs, with their N-terminal low-complexity domains, form stress granules and P-bodies that buffer modified mRNAs from degradation or transiently remove them from translation. In tumors exposed to chronic stress, altered m^6^A patterns and YTHDF expression can change which transcripts are routed into these assemblies, thereby tuning stress resilience, apoptosis sensitivity, and metabolic reprogramming [134]. YTHDF1 promotes proliferation and metastasis in a wide range of tumors [135]. For example, in ovarian cancer, YTHDF1 supports tumor growth and metastasis by augmenting EIF3C translation, and its silencing inhibits tumor progression in vitro and in vivo [24].

Recent studies provide direct evidence that YTHDF2-mediated phase separation is itself oncogenic. In arsenite-exposed keratinocytes, poly-m^6^A recognition on PTEN mRNA drives YTHDF2 condensate formation. These condensates recruit EIF2AK1, inhibit PTEN translation, and activate AKT signaling. Phase separation-defective YTHDF2 mutants blunts tumorigenicity [136]. This is similar in the case of particulate matter 2.5 (PM_2.5_), a carcinogen that is linked to increased risk of lung cancer. Exposure to PM_2.5_ leads to enhanced YTHDF2 phase separation to suppress GADD45A (growth arrest and DNA damage 45 alpha) expression in an m^6^A-dependent manner, and disruption of YTHDF2 BMCs, reducing PM2.5-induced transformation in vitro and in vivo [25]. Although the oncogenic role of YTHDF2 in AML was intensively studied, showing that it is crucial for leukemic stem cell maintenance [92], there is no direct evidence that YTHDF2’s phase separation per se is required for AML.

IGF2BPs provide another layer: they form m^6^A-dependent cytoplasmic condensates that stabilize pro-oncogenic mRNAs, and cancer-associated changes in IGF2BP phase behavior or PTMs can enhance formation of these stabilizing hubs.

#### 4.2.3. Other Modifications and the Tumor Microenvironment

Other modifications also connect BMCs to tumorigenesis. In cancer, m^1^A writers and erasers are frequently dysregulated, and m^1^A marks on mRNAs, lncRNAs and tRNA-derived fragments influence transcript stability, translation, cell-cycle control, and metabolic pathways; however, direct biophysical evidence linking m^1^A-dependent phase separation to tumorigenicity is still limited compared to m^6^A. One example is the role of the m^1^A writer TRMT6 in lung squamous cell carcinoma (LUSC) [126], where TRMT6 is strongly upregulated in LUSC and installs m^1^A on cell-cycle-associated mRNAs such as TOPBP1 and DSN1, stabilizing them by YTHDF3 binding and promoting proliferation in vitro and in vivo. Changing the m^1^A site in DSN1 by genetic manipulation induced its expression and promoted cell proliferation. Re-methylating the site by dCasRx–TRMT6 targeting recapitulated the original state [126].

Another clear example was demonstrated in ocular melanoma, where histone lactylation upregulates ALKBH3, which erases m^1^A from SP100A mRNA, and, thus, reduces YTHDF1-dependent SP100A mRNA stabilization. This lowers SP100A protein levels, attenuates PML nuclear condensates, and promotes tumor progression, establishing an m^1^A-SP100A-PML axis that directly links RNA methylation to the abundance of a tumor-suppressive BMC compartment [33].

Beyond intrinsic tumor growth, condensate-forming m^6^A readers, such as IGF2BPs, are increasingly implicated in shaping the tumor microenvironment and immune evasion, suggesting that phase-separated mRNP hubs may also modulate anti-tumor immune signaling and responses to immunotherapy, as summarized in multiple reviews (for example [92,102]). More specifically, IGF2BPs expression correlates with immune infiltration, PD-L1 levels, interferon responses and sensitivity to checkpoint blockade [137]; moreover, genetic or pharmacologic inhibition of IGF2BPs can enhance antitumor immunity and improve immunotherapy responses in preclinical models [138,139]. At the same time, biophysical and cell-biological work indicates that IGF2BPs can form mRNP condensates and phase-separated assemblies with target mRNAs [27]. However, existing data do not yet demonstrate that the phase-separation behavior of IGF2BP-condensates is the causal mechanism underlying tumor immune evasion or clinical immunotherapy resistance. Still, the concept that IGF2BP-containing condensates serve as dedicated immune-regulatory hubs—although grounded in their m^6^A-dependent stabilizing functions and immune-related target sets, and presence and role in driving BMCs formation—needs further studies.

Together, these examples illustrate that in cancer, dysregulated RNA modifications can select which RNAs act as scaffolds or clients in condensates and alter the phase behavior of reader proteins, collectively reconfiguring nuclear and cytoplasmic condensate landscapes to favor malignant phenotypes.

### 4.3. Neurodegenerative Disease: Condensate Hardening and Toxic RNP Assemblies

Neurodegenerative diseases such as Alzheimer’s disease, Parkinson’s disease, and amyotrophic lateral sclerosis (ALS) are characterized by progressive loss of specific neuronal populations, accompanied by abnormal protein and RNA assemblies that disrupt cellular homeostasis and ultimately lead to cognitive and motor decline. In such diseases, RNA modifications and phase separation intersect mainly through RNA-binding proteins that undergo aberrant condensation and maturation into aggregates. Here, modified RNAs can act as pathological scaffolds that recruit and mislocalize RBPs, toward more solid, less reversible, and more aggregation-prone states.

#### 4.3.1. m^6^A in Neurodegeneration: Correlational Links and a Mechanistic Exception

Dysregulated m^6^A and its associated proteins (writers, readers and erasers) are increasingly implicated in neurodegenerative diseases, and many of these factors have been shown to undergo phase separation in other cellular contexts. However, a direct mechanistic link where altered m^6^A drives mRNA-mediated BMC formation that in turn causes neurodegeneration has not yet been conclusively demonstrated. Many reviews discuss the evidence linking m^6^A to different neurodegenerative diseases (for example, [140,141]). m^6^A is expected to affect normal brain development and disease states since it is involved in multiple neurological processes, such as neurogenesis, synaptic formation, axonal growth and guidance, and neuronal plasticity [140,141,142]. Moreover, m^6^A-modified mRNA in the nervous system is comparatively abundant, and its dysregulation has been associated with cognitive dysfunction [141,142].

In mature circuits, m^6^A tunes synaptic protein synthesis and activity-dependent plasticity, for example through FTO and YTHDF1 at synapses [141]. With aging, m^6^A patterns and regulator expression shift in a region- and cell-type-specific manner, altering methylation of synaptic genes and contributing to stress responses, mitochondrial function, and lifespan differences between neuronal and glial compartments [140,141]. Disrupted m^6^A plays a major role in neurodegenerative and cognitive disorders—including Alzheimer’s, Parkinson, Huntington diseases—as well as metal-induced cognitive dysfunction [140], proposing that dysregulated m^6^A contributes to synaptic dysfunction, inflammatory imbalance, and disease susceptibility [140,141,142]. For example, the expression levels of the m^6^A writer METTL3, along with changes in mRNA m^6^A levels, were studied in the context of Alzheimer’s disease [143]. The magnitude of these changes and their consequences suggest that perturbation in m^6^A may impact neuronal dysfunction and disease onset, although these changes are not directly linked to phase separation in Alzheimer’s disease [143]. The clearest evidence that m^6^A can shape a disease-associated condensate comes from ALS models [144]. m^6^A appears to have a particularly strong impact on SG dynamics under ALS-like conditions. In familial ALS models expressing the cytoplasmically mislocalized FUS P525L mutant, cells exhibit globally elevated m^6^A levels together with an increased number of FUS-positive SGs and prolonged persistence after stress. Partial depletion of the m^6^A writer METTL3 lowers mRNA m^6^A by roughly 20–40%, normalizes SG number and recovery kinetics toward wild-type behavior, with minimal effects on granules formed in cells expressing wild-type FUS [144]. Conversely, METTL3 overexpression or depletion of the m^6^A eraser ALKBH5, increases SG abundance, supporting a functional relationship between elevated m^6^A levels and pathological granule persistence [144]. Furthermore, mutant FUS stress granules exhibit an altered transcript content relative to wild-type granules, which METTL3 downregulation largely restored; the restored transcripts carried an elevated proportion of m^6^A-modified RNAs and were enriched for neuronal and neurodegeneration-related functions [144]. These findings suggest that m^6^A influence is context-dependent. Thus, rather than acting as a universal requirement for SG recruitment, m^6^A may function as a context-dependent regulator of granule composition, with potential consequences in disease-associated condensates.

#### 4.3.2. m^1^A and TDP-43: A Mechanistic Example

Although the knowledge regarding m^6^A is much greater than that of m^1^A, there is a clear example of the interaction between m^1^A-modified CAG repeat RNAs, TDP-43, phase separation and neurodegeneration [32]. As discussed above, m^1^A accumulation on expanded CAG repeat RNAs enhances binding to TDP-43 and drives its transition into less dynamic condensates. In neurons, expression of m^1^A-modified CAG RNAs drives TDP-43 translocation from the nucleus to cytoplasmic condensates, and reducing m^1^A levels decreases TDP-43 translocation and cytoplasmic granule formation. These changes in TDP-43 localization and condensate material state match key features of TDP-43 proteinopathy in ALS and frontotemporal dementia (FTD), directly linking this mRNA modification (m^1^A) to phase separation and to disease-relevant TDP-43 pathology [32].

Interestingly, there is also a clear mechanistic link between m^6^A, TDP-43 and neurodegenerative diseases, showing that m^6^A status on RNAs changes how TDP-43 engages its RNA targets and how toxic it is, via m^6^A readers like YTHDF2. In ALS models, hyper-m^6^A-methylation modulates RNA binding by TDP-43 and is essential for its autoregulation and toxicity, with YTHDF2 acting as potent modifiers of TDP-43-mediated neurodegeneration [145]. Independently, biophysical and cellular studies have established that TDP-43 undergoes RNA-dependent phase separation, and aberrant transitions from liquid droplets to solid inclusions are closely tied to ALS/FTD pathology [146]. Whether altered m^6^A patterns drive disease by directly reshaping the phase behavior of TDP-43-containing condensates remains an attractive but as yet untested possibility. It is important to note that most m^6^A-neurodegeneration connections are correlational, whereas the m^1^A-TDP-43 example is mechanistic.

These observations suggest that in neurodegeneration, RNA modifications may operate as modifiers of disease course rather than primary causes: by tuning the scaffolding properties of RNAs and the condensation of aggregation-prone RBPs, they can accelerate or restrain the transition from dynamic, functional condensates to toxic, solid-like assemblies.

## 5. New Perspectives and Future Directions

### 5.1. Epitranscriptomic Codes for Condensate Targeting

The contribution of epitranscriptomic marks to BMC formation is clear for several modifications, as discussed above. The mRNA modification landscape is highly complex: transcripts can be decorated with multiple distinct or similar marks on the same molecule, each recognized by different reader proteins that channel the RNA into different fates. This combinatorial layering of modifications and readers naturally raises the question of whether cells employ an “epitranscriptomic code” that integrates these marks to specify RNA destiny in a systematic way is still not clear, although evidence for crosstalk between mRNA modifications start to emerge. Conceptually combinatorial patterns of modifications on the same transcript molecule could encode which BMC will be formed, what the material state of these condensates will be and what its function will be. Experimental evidence for crosstalk between epitranscriptomic marks has emerged in the last decade, although this field is still young and many interactions are inferred from correlation or shared machinery. An example of a crosstalk between m^6^A and m^1^A showed that m^1^A on the same mRNA accelerating transcript decay. Separate studies indicate that both m^6^A and m^1^A influence SG formation: multivalent m^6^A marks on mRNAs, together with YTHDF readers, promote their partitioning into SGs and can modulate granule stability, whereas m^1^A motifs and the TRMT6/61A methyltransferase complex are enriched in SGs and contribute to transcript protection during acute stress. However, despite this convergence on the same condensate type, there is currently no direct evidence that m^6^A and m^1^A engage in combinatorial crosstalk within SGs or other phase separation-driven compartments. Phase separation currently stands out as a context in which an “epitranscriptomic code” is hypothesized but remains largely un-decoded. While individual marks such as m^6^A and m^1^A clearly influence condensate behavior while the combinatorial logic between different modifications is only beginning to be explored. This view emphasizes that condensate formation is not governed by a simple ‘one mark–one condensate’ relationship, but rather by a combinatorial combination in which multiple RNA modifications are interpreted by cohorts of reader proteins endowed with IDRs and other low complexity domains, together with modification-induced changes in RNA structure, to collectively specify which RNAs are recruited to which condensates and how these assemblies behave. Still, the field lacks multi-modification mapping within specific condensates and synthetic systems where combinations are engineered.

### 5.2. Bidirectional Feedback: Condensates Regulating Modification Pathways

Phase separation not only responds to modifications, the writing and erasing machineries themselves are often embedded within BMCs. Illustrative examples are TRMT6/61A in SGs, or ALKBH3 effects on PML bodies, as discussed above. In such contexts, condensates can function as specialized reaction chambers where mRNA marks are written, erased, and read, thereby creating localized “epitranscriptomic microenvironments” with distinct modification dynamics and outputs. To rigorously establish this bidirectional feedback concept, several complementary layers of information are needed.

First, the catalytic activity of modification-associated enzymes should be quantitatively compared inside vs. outside BMCs, by combining spatially resolved or proximity-based proteomics with direct activity assays [147]. Such measurements would reveal whether phase separation enhances, inhibits, or otherwise modulates the activity of these enzymes relative to their behavior in a diffused cytoplasmic or nucleoplasmic state.

Second, it is important to determine whether the substrate selectivity of modification-associated proteins differs in BMCs. Experimental work, outside the field of epitranscriptomics, proved that BMCs can enhance or modulate enzymatic activity rates by altering the local environment rather than simply concentrating reactants [148]. For example, the catalytic activity of horseradish peroxidase (HRP) sharply increases inside BMCs compared with its activity in solution [149]. BMCs can also alter enzymatic activity by buffering local pH [149]. The ability of BMCs to change enzymatic activity through generating “islands” of altered physicochemical conditions supports the concept that they act as biochemical reaction chambers rather than passive storage sites. By analogy, modification-associated proteins may preferentially act on specific subsets of transcripts, sequence motifs, or structural states once they are in a given BMC, while their preferences may be different when in a soluble state.

Third, perturbing condensate integrity, through inducing dissolution, mutations that disrupt multivalency, or changes in cellular state, and global profiling of the modification landscape in the two states, may reveal whether condensate assembly and disassembly globally rewire where, when, and how epitranscriptomic marks are deployed.

Together, these approaches would test a model in which BMCs feed back onto the epitranscriptome by reshaping modification kinetics, substrate choice, and downstream RNA fates.

### 5.3. Material State Control as a Therapeutic Axis

As many disease-relevant proteins and RNAs are organized in BMCs, condensate material state itself—liquid-, gel- or solid-like—is a tractable therapeutic axis: rather than targeting protein abundance or enzymatic activity alone, interventions could shift condensates between states to alter how transcripts are stored, modified or translated [150,151].

In neurodegenerative diseases, such as ALS and FTD, aberrant phase transitions convert dynamic liquid-like assemblies into more solid, less reversible structures, locking RNAs and proteins in dysfunctional, more solid-like condensates [152]. Cancer-linked condensates frequently show dysregulated assembly or material properties, including gain or loss of specific condensates, altered composition and shifts toward either more liquid-like or more solid-like states in a context-dependent manner [151,153]. For example, mutations in the tumor suppressor SPOP disrupt its formation of nuclear condensates required for substrate ubiquitylation, leading to failed condensate assembly and accumulation of oncogenic substrates, rather than a generalized more fluid state [153]. Likewise, AKAP95 studies indicate that both loss of condensation and excessive hardening can impair gene regulation and tumorigenesis, suggesting that appropriate liquidity and dynamics are crucial for some oncogenic condensates [154]. These and other examples support an emerging view in which tuning condensate material properties, either by disrupting pathogenic condensates or restoring normal biophysical behavior, may offer therapeutic opportunities to specific tumors. Embracing the use of small molecules, peptides, or engineered scaffolds that can modulate multivalency, interaction strength, or local condition (such as pH) may, in principle, tilt these systems back toward a more physiological state [150,151,153], preventing their transition into dysregulated condensates to block their function [150].

For mRNA modification pathways, material state control opens an additional layer of intervention. Since writers, erasers, and readers often reside in condensates, altering condensate material state or composition could selectively redistribute modification-associated enzymes, change their local concentration, and rewire which transcripts they act upon (discussed in Section 3). Reducing pathological scaffolding modifications, such as m^1^A on repeat RNAs, may keep BMCs in a more dynamic, reversible state. Therapeutic strategies that tune condensate properties may, therefore, reshape the epitranscriptomic landscape without directly inhibiting catalytic sites, for example, by displacing a reader from SGs or transcriptional condensates, or by preventing a writer complex from forming a high-concentration nuclear hub.

Genetic and physiological perturbations of m^6^A writers and readers already show that these factors are required for the formation and stability of multiple condensates, including SGs, nYACs, NSs, and enhancer-associated transcriptional condensates, and that altering m^6^A pathways can reduce or remodel these BMCs. Small-molecule inhibitors of m^6^A-associated proteins are being developed, and the first-in-class METTL3 inhibitor STC-15 (STORM Therapeutics) has entered clinical trials for advanced solid tumors (Phase 1, ClinicalTrials.gov NCT05584111; now in Phase 2 in sarcoma) [155]; however, the impact of such inhibitors on condensate material properties and phase separation behavior remains to be systematically explored.

Conceptually, this frames BMCs as potential drug targets, in which instead of targeting individual molecules, therapies would target the collective material behavior of many molecules in a specific condensate. Although this potential will require tools to precisely measure and manipulate only certain condensate material properties in vivo, it offers an appealing novel route to intervene in diseases that are linked to BMC formation and are driven by dysregulated RNA metabolism and epitranscriptomic marks.

### 5.4. Systems-Level and Technological Needs

There is a major technological gap limiting our understanding how BMCs and mRNA modifications interact. Condensates are dynamic, nanoscale structures, and epitranscriptomic marks are small chemical changes on individual nucleotides. Studying both together requires tools that combine high spatial resolution, molecular specificity, and temporal control.

On the BMC side, there is a need for live-cell imaging methods that can report not only condensate location and dynamics, but also local physicochemical properties (viscosity, pH, crowding) that influence the activity of modification-associated enzymes. On the mRNA side, we need quantitative mapping of multiple modifications in situ, ideally at single-molecule or single-condensate resolution, rather than in bulk extracts.

Bridging these limitations scales calls for integrated approaches: (1) **Spatial proteomics and condensate-targeted tagging** [147] will help identify which enzymes and transcripts are present in a given BMC at a particular time. (2) **Activity reporters** for writers, erasers and readers inside condensates will uncover condensation-dependent changes. (3) **Perturbation tools** that switch the condensation process on and off. Acute dissolution strategies coupled to fast, sensitive readouts of the modification landscape are needed to shift from correlative observations to mechanistic insights. Such tools include light controlled, optogenetic systems [156,157], and small molecules that directly disrupt or enhance condensate formation [158,159,160]. Although sudden changes in temperature, salt, or crowding can dissolve particular BMCs in vitro and sometimes in cells, since these are less specific and alter the modification landscape, they are not suitable in this context. (4) **Multiomic condensate mapping** will combine data on the RNA targets of a protein of interest by tools such as CLIP-seq, modification mapping, (reviewed in [92,161]) and proteomics in purified condensate fractions to dissect the content in each BMC population [56,147]. (5) **Live-cell reporters**: Synthetic RNA reporters with different multivalent combinations of modifications, together with fluorescently labeled readers, will allow condensate recruitment and material state to be monitored in real time. Toward this approach, a recent report designed a modular versatile system that utilizes *de novo*-designed RNA motifs to engineer programmable synthetic condensates in mammalian cells, demonstrating small-molecule control of condensation ([162], a non-peer-reviewed preprint). Another study reports a related programmable RNA-condensate system in mammalian cells [163]. (6) **Quantitative biophysics:** There is a technological and conceptual need for more carefully controlled in vitro studies in which mRNPs containing a recombinant protein of interest, introduce specific, chemically defined RNA modifications at specific positions, and how those modifications affect condensation and material properties.

## 6. Concluding Remarks

BMCs are dynamic membraneless organelles composed of proteins and RNAs, whose formation and material properties are shaped by multiple factors, with mRNA modifications now recognized as important contributors that can influence when and where these structures emerge. By changing the modification landscape, cells can rewire which RNAs are recruited into particular condensates, thus, reshaping gene expression programs across health, stress adaptation, and diverse disease states. At the same time, a substantial experimental gap remains to fully resolve the intricate relationships between different epitranscriptomic marks, their crosstalk, the activities and localization of their associated writer, eraser and reader enzymes, and the detailed condensate topologies they generate, some of which reflects current technical limitations in measuring modifications and condensate dynamics with sufficient spatiotemporal and chemical resolution.

## Data Availability

No new data was created or analyzed in this study. Data sharing is not applicable to this article.
